# Focus on Rare Tracheal Pathologies You Should Not Miss

**DOI:** 10.3390/jcm15145474

**Published:** 2026-07-13

**Authors:** Anna Annunziata, Roberto Rega, Lidia Atripaldi, Antonietta Coppola, Mariano Mollica, Luigi Fiorentino, Candida Massimo, Raffaella Lucci, Antonella Marotta, Giuseppe Fiorentino

**Affiliations:** 1 Unit of Pathophysiology and Respiratory Rehabilitation, Department of Intensive Care, AORN Ospedali dei Colli, 80131 Naples, Italy; anna.annunziata@gmail.com (A.A.); lidia.atripaldi@virgilio.it (L.A.); antonietta.coppola@ospedalideicolli.it (A.C.); mariano.mollica@ospedalideicolli.it (M.M.); luigifiorentino2001@gmail.com (L.F.); austen.anto@gmail.com (A.M.); giuseppefiorentino1@gmail.com (G.F.); 2 Unit of Radiology, AORN Ospedali dei Colli, 80131 Naples, Italy; candida.massimo@ospedalideicolli.it; 3 Unit of Pathological Anatomy, AORN Ospedali dei Colli, 80131 Naples, Italy; raffaella.lucci@ospedalideicolli.it

**Keywords:** chronic cough, dyspnea, asthma, Mounier–Kuhn syndrome, tracheobronchomalacia, tracheobronchopathia osteochondroplastica, central airway disease, dynamic CT

## Abstract

**Backgroud**: Pathologies of the trachea and large bronchi are a heterogeneous group of rare structural disorders of the airway wall that are frequently underdiagnosed because their symptoms overlap with common respiratory diseases. **Methods**: We present three patients with chronic cough and dyspnea lasting approximately three years who had previously received multiple incorrect diagnoses, including COPD, asthma, gastroesophageal reflux, and acute bronchitis. **Case Description**: Following hospitalization for acute respiratory failure, comprehensive differential diagnostic workups revealed Mounier–Kuhn syndrome (tracheobronchomegaly) in Case 1, idiopathic tracheobronchomalacia in Case 2, and tracheobronchopathia osteochondroplastica in Case 3. Tracheobronchomegaly involves marked dilatation of the trachea and main bronchi; tracheobronchomalacia is characterized by expiratory airway collapse due to loss of cartilage stability; and tracheobronchopathia osteochondroplastica is a disease of unknown etiology featuring abnormal submucosal chondrification and ossification with luminal obstruction. All three impair mucociliary clearance and predispose to recurrent infections and progressive respiratory dysfunction. **Conclusions**: Through these cases we underscore the diagnostic challenges, characteristic radiological findings, and diverse management strategies of these conditions, emphasizing the critical need to consider tracheobronchial anomalies in patients with chronic respiratory symptoms and the key role of computed tomography in establishing an accurate diagnosis.

## 1. Introduction

Pathologies of the trachea and large bronchi encompass a heterogeneous and complex group of conditions that can alter airway structure or cause obstruction. These structural alterations lead to impaired mucociliary clearance, recurrent lower respiratory tract infections, and progressive respiratory dysfunction. In clinical practice, the greatest diagnostic challenge stems from their nonspecific clinical presentation [1]. Establishing an accurate diagnosis requires excluding cardiac comorbidities and performing a thorough differential diagnosis, which relies heavily on second-level investigations such as paired inspiratory–expiratory high-resolution computed tomography (HRCT) and flexible bronchoscopy [2].

We present three cases, admitted to our department during the last year; all three patients had in common a history of persistent dyspnea for more than 3 years and chronic cough. They also complained of increased susceptibility to respiratory infections (>3 times per year) with slow resolution.

## 2. Case Presentation

### 2.1. Case 1

A 70-year-old male, non-smoker, former rifleman with occupational exposure to smoke and dust, with a BMI of 20 kg/m^2^, presented with a history of dyspnea and chronic cough lasting for several years. Over the last four years, he experienced an increased frequency of respiratory exacerbations, reporting up to four episodes per year. Over the years, the patient had undergone pulmonary examinations with a diagnosis of acute bronchitis.

He had been previously misdiagnosed with chronic bronchitis and gastroesophageal reflux disease. Treatments with bronchodilators, anti-reflux medications, proton pump inhibitors and numerous courses of antibiotics had proven unsuccessful.

Allergy tests for inhalants and foods were negative. Blood chemistry results were within normal limits. Pulmonary function tests were within normal limits: FVC 3.61 L (98% of predicted), FEV1 3.37 L (120%), TLC 6.03 L (89%), RV 2.76 L (103%), and DLCO 6.22 mmol/(min·kPa) (81%). A previous chest X-ray had only shown an accentuation of the bronchial markings in the absence of further pathological findings.

Four months after his last exacerbation, the patient was admitted to the emergency department for acute respiratory failure. A chest HRCT scan performed upon admission revealed pneumonia. Importantly, it also highlighted marked tracheomegaly (transverse diameter of 30 mm), bronchomegaly (right main bronchus diameter of 21.2 mm; left main bronchus diameter of 20.3 mm), and multiple small bronchial wall diverticula, most prominently at the level of the left main bronchus (Figure 1). The maximum transverse diameter of the normal trachea is 25 mm; the maximum diameter of the right main bronchus is 20 mm and 18 mm for the left one [3].

Based on these specific radiological findings and the clinical picture, a diagnosis of Mounier–Kuhn syndrome (MKS), also known as tracheobronchomegaly, was established. Bronchoscopy was not performed as the patient rapidly improved during medical therapy with intravenous corticosteroids, acetylcysteine and broad-spectrum antibiotics. During the hospital stay, he began physiotherapy with HFNC and bronchial clearing. The patient continued medical therapy with acetylcysteine at a dose of 600 mg/day and began a day hospital rehabilitation program lasting 3 weeks every 3 months, with no further exacerbations to date, 8 months after diagnosis.

### 2.2. Case 2

A 63-year-old woman, non-smoker with no history of allergies or asthma, and a BMI of 33 kg/m^2^, presented with a 4-year history of dyspnea and chronic cough. She had undergone pneumological, otorhinolaryngological and gastroenterological examinations. Previous diagnoses of gastroesophageal reflux disease and bronchial asthma had been treated without clinical improvement.

Over the past year, her worsening dyspnea prompted a comprehensive diagnostic workup that ruled out cardiac conditions, neurological disorders, and pulmonary embolism. Allergy tests were negative. Blood chemistry and C-reactive protein results were within normal limits. Pulmonary function tests revealed: FVC 1.91 L (75% of predicted), FEV1 1.70 L (75%), TLC 3.80 L (77%), RV 1.66 L (103%), and DLCO 5.01 mmol/(min*kPa) (91%).

Last winter, she was admitted to the emergency department for acute respiratory failure, presenting with cough and wheezing. A throat swab tested positive for human rhinovirus. Upon clinical stabilization, a paired inspiratory–expiratory chest HRCT scan was performed. The expiratory phase revealed dynamic tracheal collapse, with the posterior membranous portion bowing anteriorly, creating a crescent shape in the axial plane. Crucially, the scan demonstrated a reduction of more than 50% in the anteroposterior diameter. A reduction of ≥50% in airway diameter during expiration has served as the standard diagnostic threshold for airway malacia. On expiration, the tracheal anteroposterior diameter decreased from 16 mm to 7.9 mm (Figure 2). During inspiration, the trachea maintained a lunate shape, and the main bronchi exhibited a reduction in caliber. These findings established the diagnosis of tracheobronchomalacia (TBM).

Bronchoscopy confirmed the suspected diagnosis, showing expiratory collapse and separate cartilage collapse. The patient underwent a thorough rheumatological and multidisciplinary evaluation for congenital and rare diseases. Based on blood tests, autoimmune tests, and clinical evaluation, collagenopathies and rheumatological diseases were ruled out.

Furthermore, the patient was diagnosed with obstructive sleep apnea syndrome (OSAS) associated with latent respiratory failure. Her care was managed through a multidisciplinary approach including nocturnal continuous positive airway pressure (CPAP) at a pressure of 10 cm H_2_O with a nasal interface, respiratory physiotherapy, and a weight loss program. The patient experienced significant clinical improvement following the rehabilitation program. The walking test showed improvement, with the modified Borg dyspnea scale decreasing from 9 to 4 and covering 80 m more than in the test performed at the start of the rehabilitation program.

### 2.3. Case 3

A 59-year-old male patient, former smoker of 5 packs/year, presented with a 3-year history of exertional dyspnea and a 5-month history of dysphonia. He had previously received symptomatic therapy for gastroesophageal reflux disease without clinical relief.

Blood test results were within normal limits; IgE and C-reactive protein were negative. Initial pulmonary function tests showed: FVC 3.17 L (75% of predicted), FEV1 2.59 L (77%), TLC 6.01 L (87%), RV 2.30 L (98%), and DLCO 8.52 mmol/(min*kPa) (89%). A neck and chest HRCT scan revealed multiple calcific thickenings protruding into the lumen of the main airways, starting from the immediately subglottic larynx with extensive involvement of the trachea, which appeared stenotic and dysmorphic (Figure 3).

Subsequent fiberoptic bronchoscopy confirmed a stenotic subglottic space with a lumen reduction of approximately 50%. The trachea was dysmorphic due to the presence of partly calcified whitish plaques affecting the cartilaginous rings; this finding also extended widely into the main bronchial tree. Endobronchial biopsies demonstrated mucosal inflammation, coarse calcifications, and the presence of typical cartilaginous and bony tissues (Figure 4).

These clinical, radiological, and histopathological findings were consistent with a diagnosis of tracheobronchopathia osteochondroplastica (TO).

The patient underwent Nd:YAG laser therapy to relieve tracheal and bronchial stenosis and restore airway patency. At the 3-month follow-up, spirometry showed significant improvement: FVC 3.24 L (80%), FEV1 2.70 L (84%), TLC 6.01 L (85%), RV 1.73 L (71%), and DLCO 9.54 mmol/(min*kPa) (104%). The walking test also showed marked improvement, with the modified Borg dyspnea scale changing from 10 before treatment to 3 at the post-treatment assessment. However, the patient experienced a symptomatic recurrence requiring an additional laser treatment 6 months later, and he is currently undergoing regular clinical follow-up.

## 3. Discussion

Central airway diseases represent a heterogeneous group of disorders that are frequently underrecognized due to overlapping and nonspecific clinical manifestations, most commonly chronic cough, dyspnea, and recurrent respiratory infections [1]. Among these, tracheobronchomegaly, tracheobronchomalacia, and tracheobronchopathia osteochondroplastica pose significant diagnostic challenges, often leading to delayed or incorrect diagnoses such as asthma, chronic bronchitis, or COPD [2].

Tracheobronchomegaly, or MKS, was first described in 1932 and is considered rare, although its true prevalence is likely underestimated because its symptoms are nonspecific and overlap with more common respiratory conditions [3]. MKS can exhibit a highly variable clinical course, ranging from entirely asymptomatic presentations to severe respiratory failure [3,4].

The etiology remains unclear, with both congenital and acquired mechanisms proposed. Associations with connective tissue disorders such as Ehlers–Danlos syndrome suggest a possible genetic component, although no specific gene has been identified. Histopathological findings consistently demonstrate the atrophy or absence of elastic fibers and the thinning of smooth muscle layers, supporting a structural defect of the airway wall [3,4,5]. This anatomical abnormality results in airway flaccidity, marked dilatation of the trachea and main bronchi, and dynamic collapse. Consequently, this structural weakness leads to impaired mucociliary clearance, mucus retention, and a strong predisposition to recurrent infections. The presence of tracheobronchial dilatation inherently disrupts the physiological cough mechanism, perpetuating a vicious cycle of chronic inflammation that often progresses to bronchiectasis and, in advanced cases, severe respiratory failure [3].

Despite these characteristic features, MKS remains frequently underdiagnosed. Importantly, as observed in our first case, pulmonary function tests can be misleadingly normal, emphasizing that clinical suspicion must prompt second-level imaging [3]. HRCT represents the gold standard for diagnosis, allowing accurate measurement of airway diameters and confirming the severe tracheobronchial dilatation in our patient. Alongside these measurements, HRCT and flexible bronchoscopy may further reveal the presence of tracheal or bronchial diverticula—herniations of the redundant mucosal tissue through the weakened tracheal rings—and dynamic airway collapse during expiration, which are pathognomonic hallmarks of the syndrome [2,3].

In contrast, TBM is primarily defined by the excessive expiratory collapse of the central airways due to the softening and weakness of the cartilaginous support, which causes a dynamic narrowing of the transverse or sagittal diameters of the tracheobronchial lumen. The condition is increasingly recognized in patients undergoing bronchoscopy for respiratory symptoms and in those with longstanding obstructive lung disease [6,7].

TBM in adults is typically acquired, most commonly occurring secondary to chronic inflammation, prolonged mechanical ventilation, prior tracheostomy, or underlying respiratory diseases such as COPD and asthma. However, it may also be idiopathic. True idiopathic forms are exceptionally rare and require the thorough exclusion of underlying systemic inflammatory or connective tissue disorders [6]. Interestingly, our patient (Case 2) had no history of any of these risk factors, highlighting a case of idiopathic TBM. The pathogenesis of true idiopathic TBM is increasingly explored through the lens of a primary, localized structural degeneration rather than an undetected systemic disease. Recent pathophysiological models suggest that these idiopathic forms stem from an intrinsic biomechanical failure of the tracheobronchial extracellular matrix and premature localized chondromalacia [8].

A key diagnostic challenge lies in distinguishing TBM from excessive dynamic airway collapse (EDAC), a related but distinct entity characterized by a substantial invagination of the posterior membranous wall rather than intrinsic cartilage weakness. Misdiagnosis is common, particularly in patients labeled as having refractory asthma or COPD, leading to inappropriate management [6,7,8].

Dynamic paired inspiratory–expiratory HRCT imaging of the neck and thorax, along with awake bronchoscopy, are essential for diagnosis, as static imaging may fail to detect the expiratory airway collapse. Radiologically, a reduction in the anteroposterior diameter of 50% or more during expiration is typically used as the diagnostic cut-off. Regarding clinical management, conservative therapy remains the cornerstone for symptomatic patients. In cases of significant dynamic collapse, non-invasive positive pressure ventilation, particularly continuous positive airway pressure, plays a crucial therapeutic role. CPAP acts as a physiological “pneumatic stent” by providing continuous positive end-expiratory pressure that counteracts airway collapsibility, thereby reducing the work of breathing, improving mucociliary clearance, and alleviating dyspnea [8].

Finally, tracheobronchopathia osteochondroplastica (TO) represents a distinct pathological entity, defined as a rare disease of unknown etiology characterized by abnormal chondrification and ossification of the submucosa [9,10]. This leads to the formation of multiple osteocartilaginous nodules protruding into the tracheobronchial lumen, typically sparing the posterior membranous wall [9]. While this disease often has an asymptomatic course or presents with nonspecific symptoms, it can manifest with chronic cough, dyspnea, recurrent infections, and occasionally hemoptysis, further contributing to its clinical overlap with other airway diseases [10,11].

The presence of cartilaginous nodules most frequently affects the anterolateral portion of the trachea; less frequently, it involves the bronchial tree and larynx, although our patient (Case 3) presented with extensive subglottic, tracheal, and main bronchial involvement [9]. Submucosal nodules characteristically spare the posterior membranous wall and demonstrate a beaded appearance on HRCT imaging, a key diagnostic feature [9,10,11].

TO is usually identified incidentally during bronchoscopy or imaging and is frequently missed or misdiagnosed despite advances in diagnostic techniques, contributing to diagnostic delay [10,11]. Diagnosis is primarily endoscopic, though histological examination may help exclude endobronchial amyloidosis, relapsing polychondritis, and sarcoidosis [9,10].

From a pathophysiological perspective, these three entities share a common consequence—impaired airway clearance and predisposition to infection [1]—while differing fundamentally in their structural abnormalities. MKS involves airway dilatation due to connective tissue deficiency, characterized by permanent enlargement of the trachea and proximal bronchi [3]; TBM is characterized by dynamic airway collapse due to cartilage weakness or posterior membrane invagination [6,8]; and TO is defined by rigid nodular protrusions causing luminal irregularity rather than collapse or dilatation [9,10]. These differences are critical, as they directly influence both the diagnostic approach and therapeutic strategies.

Radiological and endoscopic evaluations play crucial roles in differentiating these conditions. HRCT is essential for quantifying tracheal enlargement in MKS, demonstrating dynamic collapse in TBM, and identifying the characteristic calcified nodules of TO [2], while bronchoscopy remains indispensable for direct visualization of airway dynamics, for distinguishing EDAC from TBM [6,8], and for histopathological confirmation in TO [9,10]. Contemporary imaging protocols that include dynamic expiratory HRCT are particularly valuable for characterizing the degree and mechanism of airway collapse in TBM, allowing differentiation from static obstruction seen in MKS and TO. However, the lack of standardized diagnostic criteria and the variability in imaging protocols represent significant limitations in current clinical practice, with no universally accepted definitions for TBM severity or consensus diagnostic thresholds [2,6,8].

Therapeutically, management remains largely supportive across all three conditions, with airway clearance techniques, infection control, and pulmonary rehabilitation forming the cornerstone of treatment. In TBM, non-invasive ventilation such as CPAP may provide symptomatic relief by pneumatic stenting of the airways during expiration, while careful patient selection is essential to identify those who will benefit [6,8]. Severe cases characterized by significant dynamic collapse refractory to conservative management may require invasive interventions including airway stenting or surgical tracheobronchoplasty, though patient outcomes and long-term patency remain important considerations [6,8]. Similar interventional approaches have been described in selected cases of MKS with significant airway collapse [3]. In contrast, TO is generally managed conservatively with observation and symptomatic treatment, with most patients demonstrating stable disease on long-term follow-up. Endobronchial interventions, such as laser therapy, are reserved for patients with significant airway obstruction causing functional impairment and failing conservative management [9,10,11].

The diagnosis of tracheal disease requires careful attention from the clinician. All patients had been misdiagnosed and had been receiving unsuccessful treatments for many years. The clinical picture is nonspecific and alone does not allow for a correct diagnostic definition. Also pulmonary function tests and blood chemistry tests may be insufficient, as may performing an HRCT without expert evaluation. It is necessary to know these rare conditions in order to research and recognize them (Table 1).

## 4. Conclusions

The clinical heterogeneity of pathologies affecting the trachea and main bronchi, coupled with their nonspecific presentation, often delays diagnosis. These cases highlight the importance of considering alternative diagnoses in patients with chronic respiratory symptoms and recurrent infections, emphasizing the central role of dynamic HRCT imaging and accurate radiological evaluation.

To support timely recognition of these conditions in clinical practice, we propose a diagnostic algorithm summarizing the suspicion, first-line work-up, and imaging-based differentiation of the three entities (Figure 5).

Furthermore, the early establishment of the correct diagnosis facilitates the selection of the most appropriate therapeutic strategy tailored to the underlying disease mechanisms.

Our patients presented with persistent symptoms without an apparent cause and had received inappropriate diagnoses and treatments for many years. Physicians should pay close attention to the nature of dyspnea, whether constant or exertional, and routinely include the evaluation of tracheal and main bronchial pathologies in their differential diagnosis. A major limitation in the current understanding of these disorders is the scarcity of large-scale studies and the reliance on case reports and small case series, which hinders the development of standardized diagnostic and therapeutic guidelines. This underscores the importance of increasing clinical awareness and the continued reporting of such cases to improve the recognition and management of these rare but clinically significant airway diseases.

## Figures and Tables

**Figure 1:**
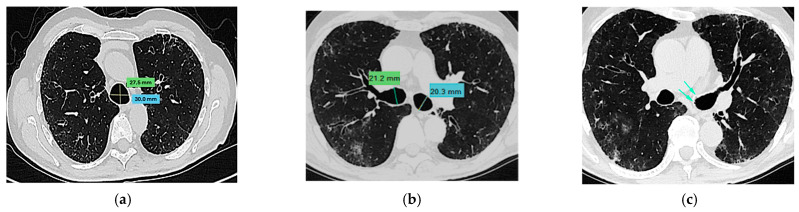
Axial chest CT of a 70-year-old male affected by MKS: (**a**) tracheal measurements showing a transverse diameter of 30 mm (blue rectangle); (**b**) right main bronchus diameter is 21.2 mm and left main bronchus diameter is 20.3 mm; (**c**) small diverticula of the bronchial wall (green arrows).

**Figure 2:**
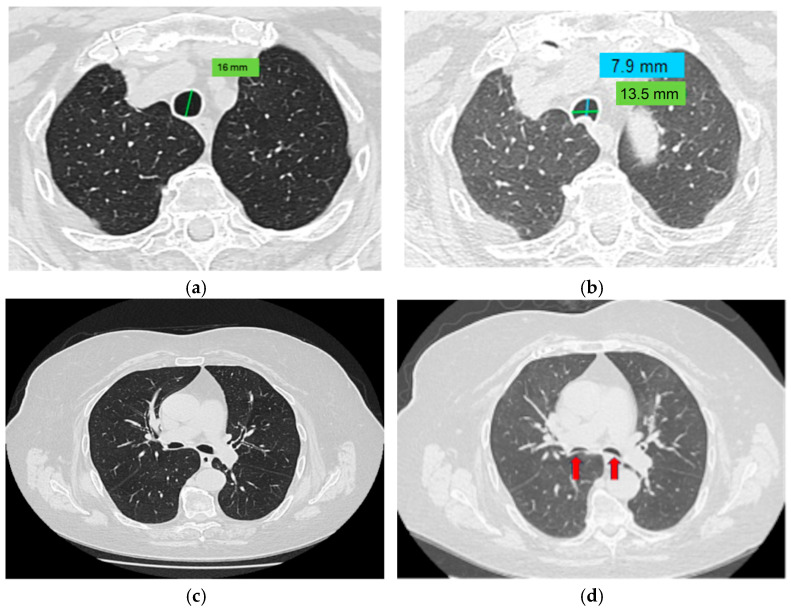
Dynamic (paired inspiratory–expiratory) axial CT of the thorax, acquired at end-inspiration (**a**,**c**) and during forced expiration (**b**,**d**). Panels (**a**,**b**) show the trachea at the same level; panels (**c**,**d**) show the main bronchi at the same level. On expiration, the tracheal anteroposterior diameter decreases from 16 mm (**a**) to 7.9 mm (**b**) (blue line), in the absence of extrinsic compression. Red arrows in panel (**d**) indicate the expiratory collapse of the main bronchi.

**Figure 3:**
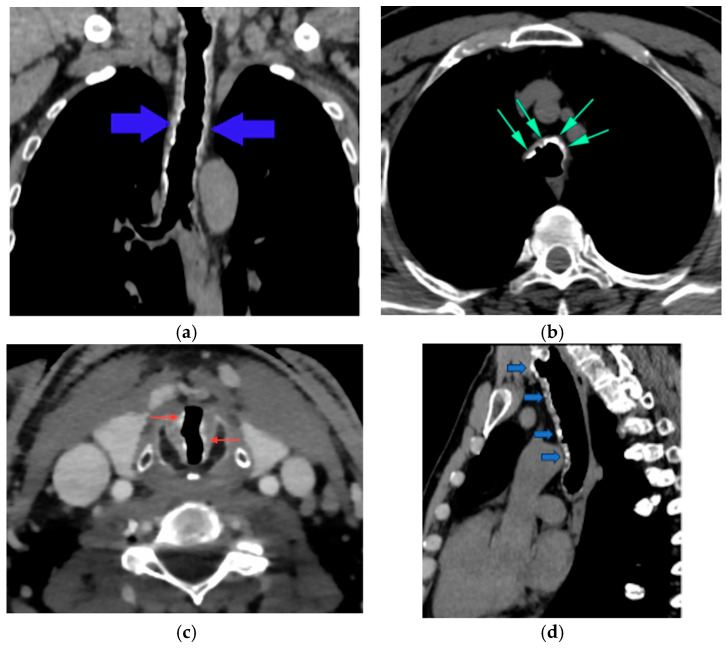
Multiplanar reconstruction (MPR) CT images showing multiple subcentimetric submucosal nodules, some of them calcified, at different levels of the trachea: (**a**) coronal plane (blue arrows); (**b**) axial plane (green arrows); (**c**) nodules at the subglottic larynx (red arrows); (**d**) sagittal view demonstrating sparing of the posterior membranous wall of the trachea (blue arrows).

**Figure 4:**
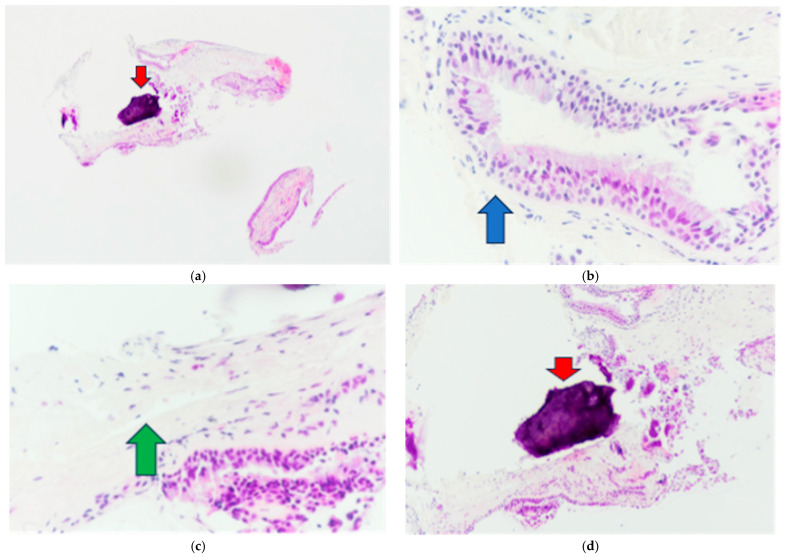
Histological features of tracheobronchopathia osteochondroplastica: bronchial mucosa lined by epithelium ((**a**) H&E ×4, red arrow), with mild squamous metaplasia ((**b**) H&E ×40, blue arrow), submucosal fibrosis ((**c**) H&E ×40, green arrow), and coarse calcifications ((**d**) H&E ×10, red arrow).

**Figure 5:**
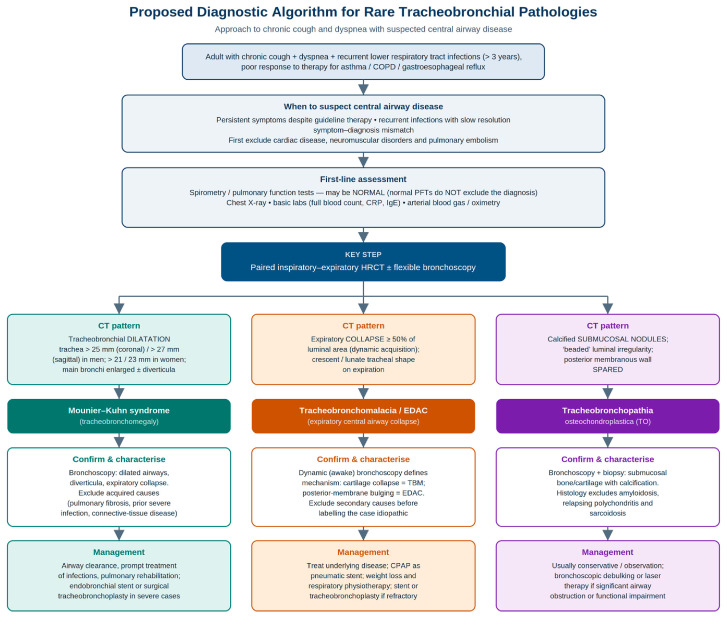
Proposed diagnostic algorithm for the three rare tracheobronchial pathologies, outlining the approach to chronic cough and dyspnea with suspected central airway disease. The algorithm is a proposed practical approach derived from the three reported cases and is not a validated guideline.

**Table 1:** Comparative summary of the three rare tracheobronchial pathologies.

Feature	Mounier–Kuhn Syndrome (MKS)	Tracheobronchomalacia/EDAC	Tracheobronchopathia Osteochondroplastica (TO)
Core abnormality	Permanent dilatation of the trachea and main bronchi (atrophy of elastic fibers, thinning of smooth muscle), causing airway flaccidity.	Excessive expiratory collapse of the central airways: cartilage weakness (TBM) or invagination of the posterior membranous wall with preserved cartilage (EDAC).	Submucosal osteo-cartilaginous nodules projecting into the airway lumen.
Etiology	Usually congenital (± connective-tissue disorders); acquired forms must be excluded.	Mostly acquired (chronic inflammation, prolonged ventilation, COPD/asthma); idiopathic is a diagnosis of exclusion.	Unknown.
Key clinical clues	Chronic productive cough, recurrent infections, bronchiectasis; may be asymptomatic.	Exertional dyspnea, barking cough, difficulty clearing secretions, recurrent infections.	Chronic cough, exertional dyspnea, recurrent infections; occasional hemoptysis.
Pulmonary function tests	Often normal or obstructive; normal results do not exclude the diagnosis.	Normal or obstructive; expiratory flow oscillations on the flow–volume loop.	Normal, or obstructive when the airway is stenosing.
CT findings	Dilatation of the trachea and main bronchi (whole circumference); tracheal/bronchial diverticula and bronchiectasis.	Paired inspiratory–expiratory CT shows excessive expiratory luminal collapse (crescent/lunate shape); posterior wall bows inward in EDAC.	Calcified submucosal nodules with irregular, narrowed lumen; posterior membranous wall spared.
Bronchoscopy & histopathology	Dilated, flaccid airways with diverticula and dynamic expiratory collapse; histology rarely needed.	Dynamic bronchoscopy confirms expiratory collapse and separates cartilage collapse (TBM) from posterior-membrane bulging (EDAC); histology not required.	Hard whitish nodules sparing the posterior wall; biopsy is diagnostic (submucosal bone/cartilage with calcification).
Differential diagnosis	Bronchiectasis of other cause, COPD.	Refractory asthma/COPD, vocal-cord dysfunction.	Amyloidosis, relapsing polychondritis, sarcoidosis.
Management	Airway clearance, infection control, pulmonary rehabilitation; stent or tracheobronchoplasty if severe.	Treat the underlying disease; CPAP as a pneumatic stent; weight loss and physiotherapy; surgery if refractory.	Usually conservative; bronchoscopic debulking or laser if the airway is obstructed.

## Data Availability

The data presented in this study are available on request from the corresponding author.

## Abbreviations

The following abbreviations are used in this manuscript:

COPD	Chronic Obstructive Pulmonary Disease
HRCT	High-Resolution Computed Tomography
FVC	Forced Vital Capacity
FEV1	Forced Expiratory Volume in 1 s
TLC	Total Lung Capacity
RV	Residual Volume
DLCO	Diffusing Capacity of the Lungs for Carbon Monoxide
MKS	Mounier–Kuhn syndrome
TBM	Tracheobronchomalacia
TO	Tracheobronchopathia Osteochondroplastica
EDAC	Excessive Dynamic Airway Collapse
CPAP	Continuous Positive Airway Pressure
CBC	Complete Blood Count
CRP	C-Reactive Protein
IgE	Immunoglobulin E
PFT	Pulmonary Function Test
HFNC	High-Flow Nasal Cannula
